# The Formation of RNA Pre-Polymers in the Presence of Different Prebiotic Mineral Surfaces Studied by Molecular Dynamics Simulations

**DOI:** 10.3390/life13010112

**Published:** 2022-12-30

**Authors:** Alix Dujardin, Sebastian Himbert, Ralph Pudritz, Maikel C. Rheinstädter

**Affiliations:** 1 Department of Physics and Astronomy, McMaster University, Hamilton, ON L8S 4M1, Canada; 2 Origins Institute, McMaster University, Hamilton, ON L8S 4M1, Canada

**Keywords:** origins of life, non-enzymatic RNA polymerization, prebiotic mineral surfaces, molecular dynamics simulations, hydrogen-bonded RNA pre-polymers

## Abstract

We used all-atom Molecular Dynamics (MD) computer simulations to study the formation of pre-polymers between the four nucleotides in RNA (AMP, UMP, CMP, GMP) in the presence of different substrates that could have been present in a prebiotic environment. Pre-polymers are $C3^{\prime }$–$C5^{\prime }$ hydrogen-bonded nucleotides that have been suggested to be the precursors of phosphodiester-bonded RNA polymers. We simulated wet–dry cycles by successively removing water molecules from the simulations, from ~60 to 3 water molecules per nucleotide. The nine substrates in this study include three clay minerals, one mica, one phosphate mineral, one silica, and two metal oxides. The substrates differ in their surface charge and ability to form hydrogen bonds with the nucleotides. From the MD simulations, we quantify the interactions between different nucleotides, and between nucleotides and substrates. For comparison, we included graphite as an inert substrate, which is not charged and cannot form hydrogen bonds. We also simulated the dehydration of a nucleotide-only system, which mimics the drying of small droplets. The number of hydrogen bonds between nucleotides and nucleotides and substrates was found to increase significantly when water molecules were removed from the systems. The largest number of $C3^{\prime }$–$C5^{\prime }$ hydrogen bonds between nucleotides occurred in the graphite and nucleotide-only systems. While the surface of the substrates led to an organization and periodic arrangement of the nucleotides, none of the substrates was found to be a catalyst for pre-polymer formation, neither at full hydration, nor when dehydrated. While confinement and dehydration seem to be the main drivers for hydrogen bond formation, substrate interactions reduced the interactions between nucleotides in all cases. Our findings suggest that small supersaturated water droplets that could have been produced by geysers or springs on the primitive Earth may play an important role in non-enzymatic RNA polymerization.

## 1. Introduction

One of the most challenging questions about the origin of life is in what environment primitive life may have started. It has been suggested that life can form near hydrothermal vents [1,2,3,4,5,6,7], in warm little ponds [8,9,10,11,12,13,14,15,16,17,18,19,20,21], or in ice [22,23,24,25]. In all these scenarios, biomolecules are in contact with substrates. Minerals are known to be catalysts for many reactions, such as molecular synthesis, selection, protection, concentration, templating or even organization of biomolecules [26]. During the Hadean period (about 4 Gy ago), more than 420 different types of minerals were estimated to be distributed on Earth [26]. The question is what mineral potentially helped the formation of life, and what kind of interactions between biomolecules and minerals are important for the emergence of life? To answer those questions, numerous experiments included minerals, whether to understand how chirality of molecules started [27,28,29,30,31], to understand the synthesis of the first building blocks of life [2,32,33,34,35,36,37,38,39,40,41,42], the first metabolism [1,43,44,45,46,47,48,49,50,51,52,53,54,55,56], the formation of primitive cells [57,58,59,60], to synthesize peptides [60,61,62,63,64,65], or to synthesize ribonucleic acids (RNA) [17,60,64,66,67,68,69,70,71,72,73,74,75,76,77].

RNA synthesis becomes particularly relevant since primitive life is thought to have started with RNA because RNA can store genetic information and it is also a catalyst [78,79,80,81,82]. RNA is also capable of evolution in vitro [83,84,85,86,87,88,89]. Modern RNA is mainly synthesized via RNA-polymerases by using DNA as a matrix. However, it is not clear how such molecules could have been synthesized 3.5 billion years ago. Since the discovery of the catalytic activities of RNA, many scientists have addressed the question of RNA synthesis in the prebiotic world [90]. Finding a catalyst/template to promote RNA synthesis is essential since it is thermodynamically not possible for polymerization to happen in water only.

Minerals are likely important for the origin of life research for multiple reasons. One of the main benefits of the minerals is to concentrate biomolecules in solution. It has been shown in many studies that charged surface minerals adsorb charged biomolecules. For example, Ferris and Ertem [72,91] highlighted that the protonated base and the negative charges of montmorillonite surface interact via electrostatic interactions. Franchi et al. [92] have shown the importance of the type of cation used for binding to the surface. They exposed that divalent cations were better at mediating the adsorption compared with the monovalent cations. Other substrates have been used to concentrate nucleotides [93]. It is, however, possible to concentrate biomolecules in water without surfaces. Mast et al. [94] conducted experiments on thermal gradients that led to the formation of polymers due to a thermal trap. They explained that the thermal trap increases the sequence space availability and induces active catalytic polymer formation.

As RNA synthesis from monomers is thermodynamically unfavorable in the presence of water, many of these studies in aqueous environments used activated nucleotides. In this study, we investigated the interaction between non-activated nucleotides and nine substrates that have been suggested to be present on primitive Earth [26] using all-atom Molecular Dynamics (MD) simulations. Water molecules were successively removed during the simulations to mimic dehydration. Three clays were investigated (montmorillonite, kaolinite, and pyrophosphate), one mica (muscovite), one phosphate mineral (hydroxyapatite), one silica (quartz), two metal oxides (corundum, and periclase), and one carbonaceous material (graphite).

**Figure 1:**
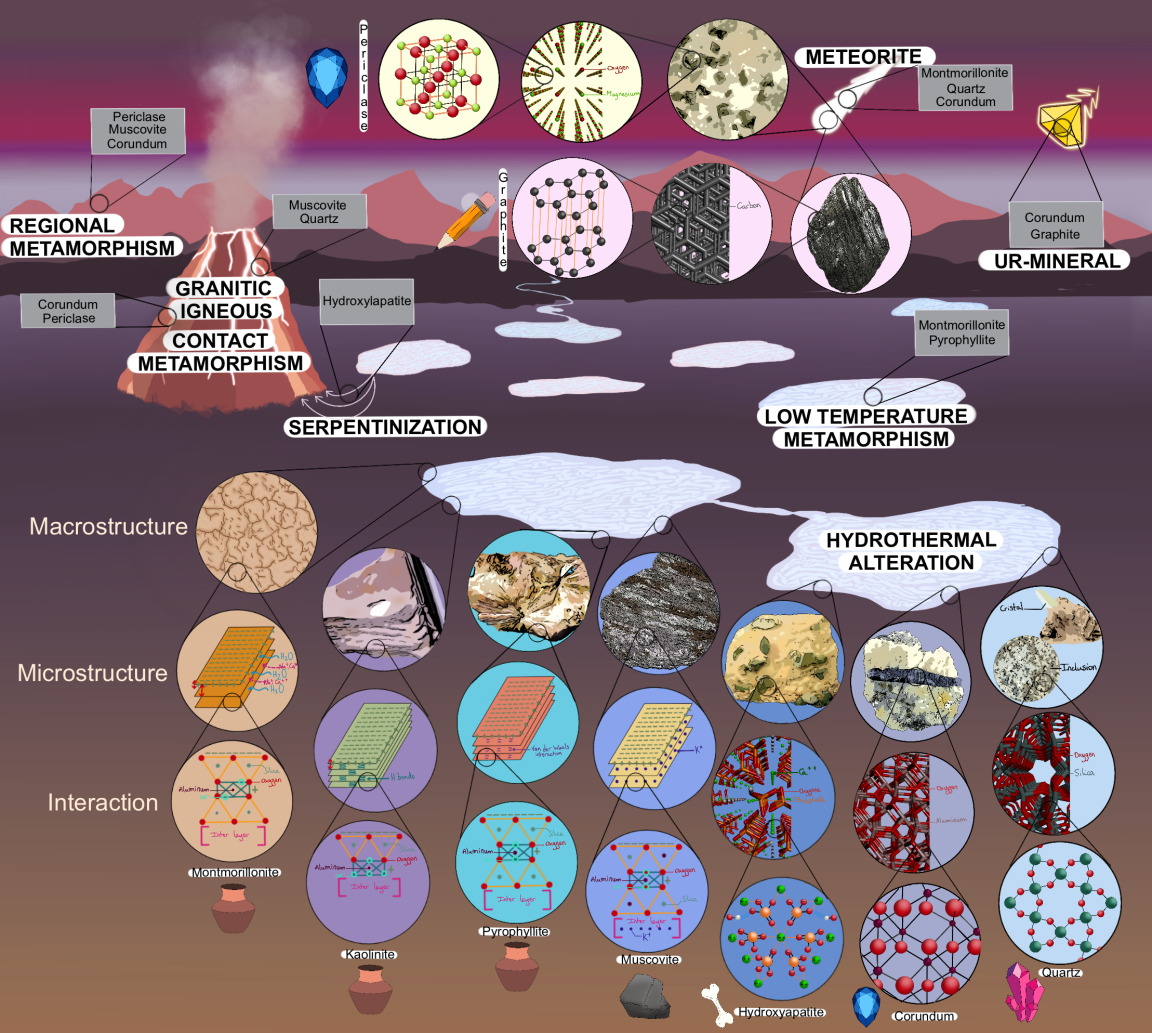
Sketch of primitive Earth including the nine substrates investigated in this study. Each substrate differs in its macrostructure, microstructure, and interaction between the atoms. They occur in different environments, such as hydrothermal alteration, low-temperature metamorphism (where pressure is higher), regional metamorphism (large-scale action of heat and pressure), contact metamorphism (contact with or proximity to a magmatic rock), serpentinization (contact between seawater and magmatic rock with a low silica rate), meteorite, in granitic igneous rocks (specific magmatic rocks), and in Ur-mineral (minerals formed from presolar grains) [26]. Clay substrates are represented by a jar (montmorillonite, kaolinite, pyrophyllite), mica is represented by a rock (muscovite), the phosphate mineral by bone (hydroxyapatite), metal oxides by sapphire (corundum, and periclase), the crystal represents the silicate mineral (quartz), and the pencil represents the carbonaceous material (graphite). Representations are used throughout the manuscript.

These nine substrates were chosen because of their interest in origin of life research. Clays are known to have reactive surfaces, high cations exchange, and catalytic activities in aqueous environments [95] and clays are often used in origin of life research because of these properties [45,46,57,66,67,68,69,70,71,72,93,96,97,98]. Montmorillonite and kaolinite are abundant clays on Earth [99,100]. We also included pyrophyllite, which has aluminum atoms sandwiched between two silica sheets, and kaolinite, which consists of a single sheet of silica, only. All three clays can be found where hydrothermal alteration occurred, such as in warm little ponds or in epipelagic environments, as sketched in Figure 1. Montmorillonite and pyrophyllite are found at low-temperature metamorphism, and montmorillonite can also be present in meteorites [26]. Muscovite is a common mineral on Earth, which can be compared to clays because it has a similar structure to montmorillonite. It is authigenic (formed where it is found), but it can also be found in granitic igneous, and where hydrothermal alteration and regional metamorphism occurred. Some traces are found in Hadean zircons (the oldest mineral that survived Earth’s transformation) [26,101]. Some scientists used muscovite as a “primitive cell” because it has common points with our modern cells [102,103,104,105].

Hydroxyapatite is the main constituent of dental enamel, dentin, and bones. It is found where hydrothermal alteration and serpentinization happened [26,74]. Hydroxyapatite was included in this study because it was used in several experiments for phosphate and nucleotide synthesis linked to the origin of life [38,39,74]. Silicate minerals (quartz) represent more than two-thirds of the Earth’s crust. As a result of their abundance on Earth, silica was one of our minerals selected. They can be found in granitic igneous, where hydrothermal alteration occurred, in meteorites and clastic sedimentary environment, as well as in Hadean zircons inclusion [26,95]. Quartz is used in origin of life research related to the chirality of molecules [27,28,29,30].

Corundum has, among others, two gem varieties: Ruby and sapphire. It is commonly found in meteorites but it can also be found in alkali igneous (in the basic and volcanic environments), where contact and regional metamorphism happened, in hydrothermal alteration, in a clastic sedimentary environment, and Ur-minerals from pre-solar grains. This mineral was used to understand the formation and the stability of the first cells [106,107]. Periclase is usually found in marble produced by contact, and regional metamorphism in the form of inclusion, as well as in meteorites [26]. Periclase and corundum were chosen because these minerals can be found in small inclusions in bigger rocks. Their atoms’ organization provides them a hardness that is not present in the other minerals studied. Graphite is one of the presolar dust grains [108], but it can also be found in metamorphic rocks. Some researchers used it to understand the first metabolism [109] and the concentration of nucleic bases [110,111].

In the wet–dry cycles hypothesis for the origin of life [15,16], nucleotides are mixed in an aqueous phase and then dried on a surface. At the same time, the available thermal energy for sufficiently warm conditions drives the formation of a phosphodiester bond between the $C3^{\prime }$ and $C5^{\prime }$ of neighboring nucleotides. This assumption implies that nucleotides align in a way that brings these two atoms in close proximity when they dry out on a surface. The question that we address is to what extent various mineral substrates are able to promote polymerization by either securing favorable proximity or securing favorable molecular arrangements of the nucleotides. We address this question by conducting a comprehensive series of MD simulations in which we carefully follow the dynamics of nucleotides in their interaction with one another and as well as the nine surfaces of minerals noted in Figure 1 as dehydration takes place. In particular, we track the appearance of hydrogen bonds between the $C3^{\prime }$ and $C5^{\prime }$ atoms that are probably precursors of covalent bond formation. These nucleotides, which are aligned, however, have not yet formed phosphodiester bonds have been named ‘pre-polymers’ [9]. These pre-polymers are thus in the right conformation and ready to bond; however, ester bond formation is a rate-limiting process, unlike hydrogen bonds. Even though the formation of phosphodiester bonds using wet–dry cycles is a thermodynamically favorable process [15], the formation rate of these bonds must be as fast as possible due to numerous exterior factors that can damage RNA polymers, such as hydrolysis, pH, or temperature. Our simulations suggest that surfaces may play a secondary role in this pre-polymerization phase. It is most likely that the stage leading to polymerization in such dehydration conditions occurs in the volume of drastically dehydrated ponds and, in particular, in small droplets that are nearly completely dehydrated.

## 2. Results

### 2.1. Substrate Properties: Charge and Hydrogen Bond Potential

In the following, we classify the substrates used in this study based on two physical properties, which we believe are important for their interaction with nucleotides: (1) their charge and (2) their potential to form hydrogen bonds (Hbonds) with the nucleotides.

#### 2.1.1. Charges

**Figure 2:**
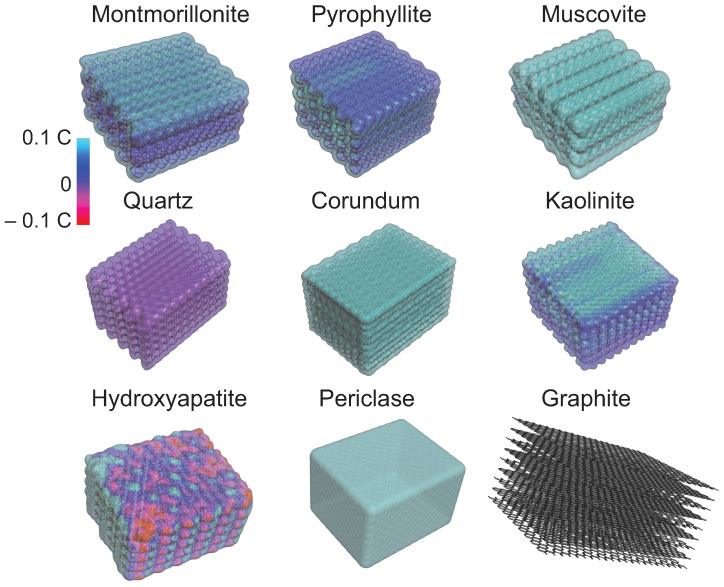
Surface map of interpolated charges for the different substrates. Light blue represents a positive charge, while red shows a negative charge. Charges vary between −0.1 and +0.1 C. Dark blue/purple illustrates a neutral charge. Montmorillonite, muscovite, corundum, kaolinite, and periclase are positively charged. Pyrophyllite and quartz are neutral. Hydroxyapatite is both positive and negative. Graphite has an inert surface. Maps were created using BIOVIA Discovery Studio 2021 [112].

The type of atoms and their arrangement determine the surface charge of a substrate. This property determines what types of molecules or molecular groups would be attracted through electrostatic interactions. To better understand the surface charge of each substrate, an interpolated charge surface map was created and is shown in Figure 2. This map was generated using the software BIOVIA Discovery Studio 2021 [112]. A partial charge is assigned to each atom on the surface. If there is no partial charge available, the system calculates the Gasteiger charge which is determined on the basis of electronegativity equilibration [113]. The limits used are −0.1 to +0.1 C. The map is then created using a numerically mapped color spectrum. In Figure 2, the light blue represents a positive surface, the dark-purple blue is associated with a neutral surface, and the red color shows a negative surface.

From these calculations, three of the substrates are strictly positively charged: muscovite’s surface is positive due to the potassium ions on the surface. Corundum is positively charged throughout its entire surface due to the aluminum present. Periclase’s overall charge is positive due to the presence of magnesium.

The montmorillonite surface tends to be positively charged but less than +0.1 C. The extremity of the folia is, however, neutral. Kaolinite tends to have similar charges as montmorillonite; however, its surface tends to be more positive due to the presence of hydrogen atoms. Hydroxyapatite’s surface is both positively and negatively charged. The oxygen attached to the phosphate gives a negative charge, while the calcium provides a positive charge. Pyrophyllite’s surface tends to be neutral due to the position of the hydroxide ions in the substrate. Quartz is also neutral because each oxygen is linked to a silica atom. Graphite is included here as a reference for a non-interacting substrate because it has no charges. The role of this substrate will be discussed further below.

#### 2.1.2. Hydrogen Bond Potential

**Figure 3:**
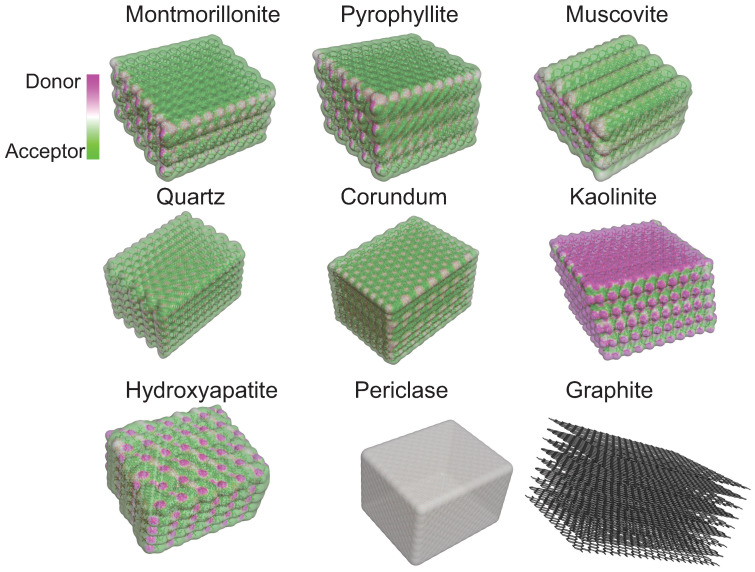
Surface map representing the substrates’ capacity to form hydrogen bonding with other molecules. In green, the substrates tend to function as hydrogen bond acceptors, while the magenta color shows the surface which functions as hydrogen bond donors. White illustrates a neutral surface. Montmorillonite, pyrophyllite, muscovite, quartz, and corundum are acceptors. Kaolinite is a donor. Hydroxyapatite is both donor and acceptor. Periclase is neutral, and graphite is inert. Maps were created using BIOVIA Discovery Studio 2021 [112].

A substrate’s capacity to form Hbonds with external molecules, such as the nucleotides, is an important physical property. The surface map in Figure 3 gives information on the area of the surface where nucleotides can bond. To do so, a map was created to extrapolate the potential donor or acceptor of each surface using BIOVIA discovery Studio 2021 [112]. Hydrogen atoms were added if needed to ensure a consistent surface. If an atom is considered as a donor, a fictitious charge *q* is given of +1 C. A fictitious charge *q* of −1 C is assigned for atoms considered acceptors. Other atoms are not taken into consideration. The charges are used to calculate a single-valued “potential” function at the location of each surface vertex. The potential is obtained as the weighted average $\sum_{q}w\left(q\right){}^{*}q$ of all charges *q*, with the weight w(q) for an individual charge *q* set to the ratio s(q)/s, where s(q) is the inverse of the square distance between the position of the surface vertex and the position of the atom carrying charge, and $s=\sum_{q}s\left(q\right)$ is the sum of the contributions *s(q)* from all charges. This potential is then mapped using a numerically mapped color spectrum as well. In this figure, green represents an acceptor surface, the magenta color represents a donor surface, and white is neutral.

Following this analysis, five substrates have the potential to form Hbonds. Montmorillonite clay surface has the potential to accept hydrogen bonds due to oxygen on the surface. However, due to the hydrogen present at the aluminum layer’s extremity, the folia’s edge functions as a donor. Thus, magenta is seen in between each repeating unit. As montmorillonite, pyrophyllite will tend to form a hydrogen bond by accepting an electron on its surface. Muscovite’s surface has the potential to accept electrons to make hydrogen bonds due to the presence of oxygen. Quartz’s overall surface functions as a hydrogen bond acceptor due to the oxygen present in the mineral. Corundum will tend to receive electrons because not all oxygens are fully connected with aluminum.

Kaolinite’s surface is the only one to work as a potential hydrogen bond donor. This is due to the presence of hydrogen linked to oxygen which made the bond between the silica atoms. At the extremity of the folia, oxygen is exposed; thus, this part of the surface is a hydrogen bond donor. The hydroxyapatite surface has both donor and acceptor properties. The oxygen ions are acceptor, while the hydrogen linked to the oxygen serve as donors. Periclase does not accept or give hydrogen because all oxygen is linked with a magnesium ion.

**Figure 4:**
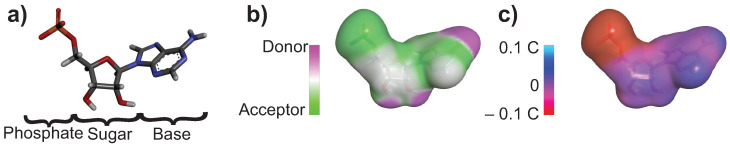
Example of a nucleotide used in the simulations. (**a**) Adenosine monophosphate (AMP) molecule. (**b**) Map of the hydrogen bond potential of AMP. (**c**) Map of the charge distribution of AMP.

Charge distribution and the potential to form Hbonds were also created for the nucleotide molecules. In Figure 4a, adenosine monophosphate (AMP) is shown as an example. Figure 4b shows the potential Hbonds surface map. The nucleotide’s atoms do not have the same capacity to form Hbonds throughout the molecule. The phosphate of the nucleotide is a hydrogen bond acceptor, while the hydroxyl on the $C2^{\prime }$ and $C3^{\prime }$ are donors as well as the amine group on the base. The other atoms of the molecules are neutral. Figure 4c shows the surface charge of the AMP molecule. Nucleotides have heterogeneous charges throughout the molecule. The phosphate of the nucleotide is negatively charged, while the other parts of the nucleotides tend to be more neutral. This figure gives information on which part of the nucleotide can potentially form electrostatic interactions and hydrogen bonds with the different substrates.

### 2.2. Molecular Dynamics Simulations

All-atom MD simulations were performed using GROMACS 2016.3 on MacSim, a GPU-accelerated computer workstation [114]. Each simulation was set up using CHARMM-GUI [115,116,117]. The size of the simulation box was 85 Å${}^{3}$. All systems contained 200 nucleotides (50 nucleotides of AMP, UMP, CMP, and GMP, respectively), the substrate, and water molecules. The system was first energy minimized and then equilibrated. Hydration was reduced in 10 steps from ~12,500 water molecules to ~600 water molecules in total, equivalent to a dehydration from ~62 to ~3 water molecules per nucleotide. Each simulation’s total production simulation time using this dehydration script was ~200 ns, resulting in a total production simulation time of about 2 μs of all-atom MD simulations for this study. More details can be found in Section 5.

**Figure 5:**
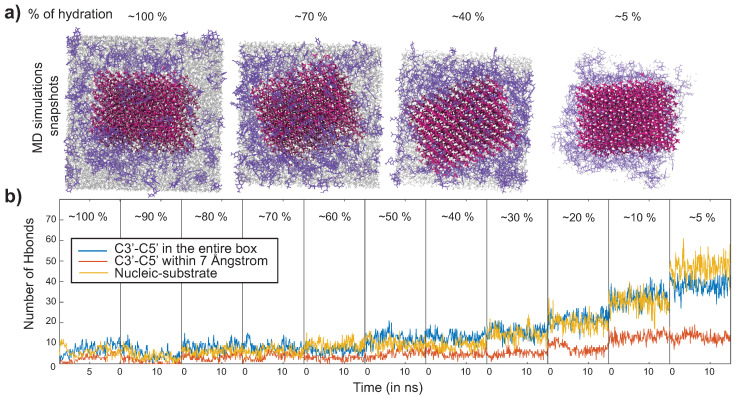
MD simulation results for hydroxyapatite as an example. (**a**) Snapshot of MD simulations at ~100%, ~70%, ~40%, and ~5% hydration. The simulation contained 200 nucleotides. Hydroxyapatite is shown in pink, nucleotides in purple, and water in gray. (**b**) Number of hydrogen bonds (Hbonds) between the $C3^{\prime }$ and the $C5^{\prime }$ of the nucleotides in the entire simulation box (blue); between the nucleotides’ $C3^{\prime }$ and the $C5^{\prime }$ within a distance of 7 Å to the substrate (orange), and between the nucleotides and the substrate (yellow), as a function of hydration. While the number of hydrogen bonds nucleotides is small (~5) when fully hydrated (at 100% hydration), bonding significantly increases during dehydration.

Figure 5a shows snapshots of the MD simulation run in the presence of hydroxyapatite. Hydroxyapatite is represented in pink, nucleotides in purple, and water in gray. Snapshots were taken at four different hydration levels: ~100%, ~70%, ~40%, and ~5%. These frames were taken at 10 ns at ~100% and 15 ns for the last three. The figure shows that every time water molecules are removed from the system, nucleotides move closer to the substrate until they saturate the substrate surface at ~5% hydration.

We used this MD simulation also to calculate the number of Hbonds during dehydration. Hydrogen bond analysis was performed using Visual Molecular Dynamics algorithms [118]. Three different interactions are plotted in Figure 5b: the Hbonds between $C3^{\prime }$ and $C5^{\prime }$ of the nucleotides in blue, the Hbonds between $C3^{\prime }$ and $C5^{\prime }$ of the nucleotides within a 7 Å distance from the substrate are represented in orange, and the Hbonds between the nucleotides (non-specific atoms) and the substrate in yellow. All substrates resulted in similar profiles, with the number of Hbonds significantly increasing when water molecules were removed from the system, resulting in denser packing and increased substrate interaction. When fully hydrated, all types combined the number of Hbonds was found to be low (~5 on average). When decreasing the number of water molecules, the number of Hbonds started to increase in all cases. A number of hydrogen bonds of ~50 at the lowest hydration of 5% then means that 25% of the nucleotides have formed hydrogen bonds (out of 200 nucleotides in total). We will focus on the results at ~5% hydration in the following, representing the fully dehydrated state.

### 2.3. Substrates with Positive Charges Attract the Nucleotides’ Phosphate Group

**Figure 6:**
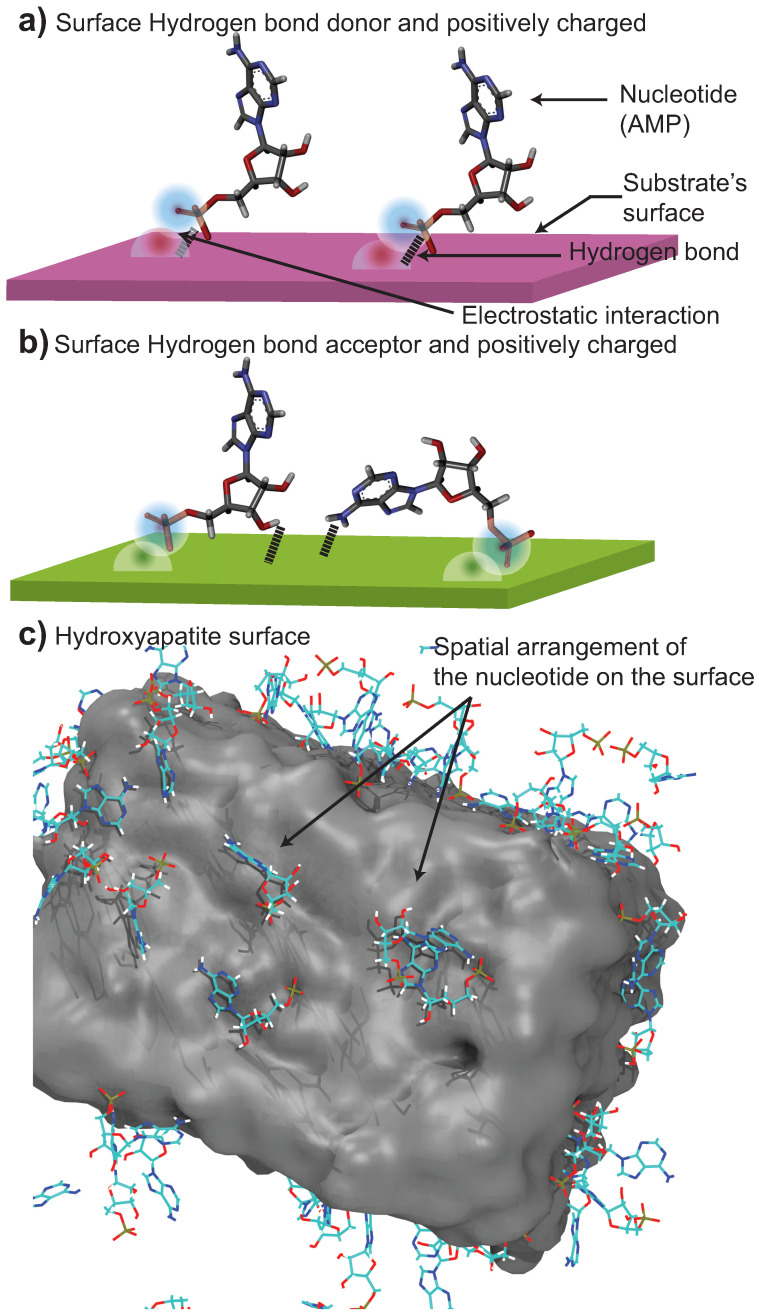
Sketch of the organization of nucleotides on the different substrates. (**a**) When the surface is a hydrogen donor and positively charged, the negatively charged phosphate is attracted through electrostatic interactions and will form Hbonds with the surface. (**b**) If the surface is an acceptor and positively charged, the phosphate will be attracted; however, the hydroxyl part of the sugar and the base will form Hbonds with the surface. (**c**) Screenshot of the hydroxyapatite MD simulation at ~5% at 15 ns showing the nucleotide conformations, sketched in (**a**,**b**). Only AMP molecules are shown for clarity.

The surface charge of the substrates determines the orientation and the attraction of the nucleotides to the surface. Different parts of the nucleotides will be attracted by electrostatic interactions and will interact with the substrate. In our study, five substrates have a positively charged surface, pyrophyllite and quartz are neutral, and hydroxyapatite has both positive and negative charges. A representation of the nucleotide orientation on the different surfaces is pictured in Figure 6. A positive surface will attract the negatively charged phosphate group of the nucleotides due to their opposite attraction (Figure 6a). Then, depending on the Hbonds potential of the surface, the nucleotides would be able to form hydrogen bonds with different parts of the molecule (Figure 6b). The donor surface will form Hbonds with the phosphate of the nucleotides because of the oxygen present. The hydroxyl on the sugar and the hydrogen on the base will form Hbonds with an acceptor surface because of their donor property. Figure 6c shows a snapshot of the hydroxyapatite MD simulation at ~5% showing the nucleotide conformations equivalent to the cartoons in parts a and b. In this figure, only AMP molecules are shown for clarity.

### 2.4. Substrates form Hydrogen Bonds with Different Parts of the Nucleotides

**Figure 7:**
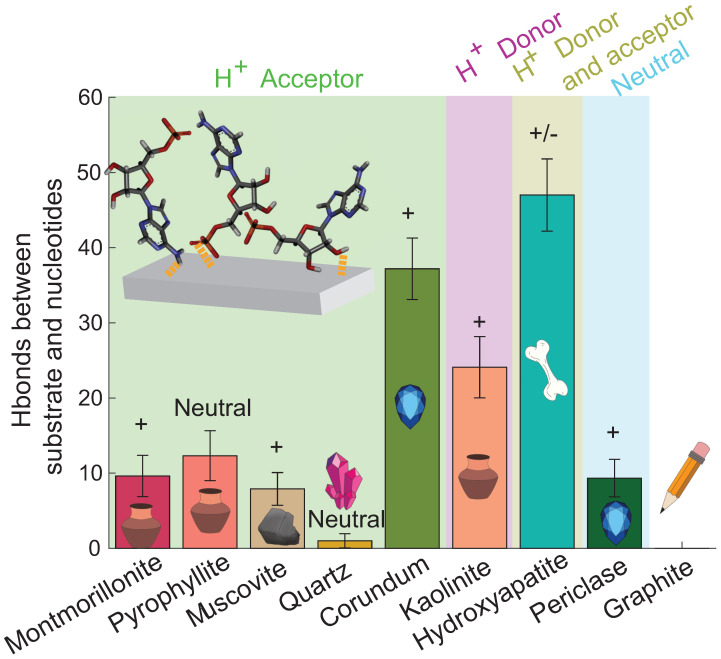
The average number of Hbonds formed between the substrates and the nucleotides at ~5% hydration. The error bars represent one standard deviation. The color blocks represent the substrates’ potential as donors or acceptors. The “+” and “−” represent the positive and negative surface charges, respectively. The largest number of Hbonds are observed for corundum, kaolinite, and hydroxyapatite.

We calculated the number of Hbonds between the nucleotides (all atoms combined) and the different substrates. Figure 7 shows the average number of Hbonds formed at ~5% hydration. Error bars represent one standard deviation. It is evident that the different substrates bond very differently with the nucleotides. Three of the four acceptor substrates do not form a significant amount of Hbonds with the substrates, as seen in Figure 7 (montmorillonite: ~10; pyrophyllite: ~12; muscovite: ~8). Corundum is an exception and it formed ~37 Hbonds with the nucleotides. Kaolinite, which is a hydrogen donor, formed ~24 Hbonds with the nucleotides. Hydroxyapatite was the most efficient for forming Hbonds with the nucleotides by having both hydrogen donor and acceptor atoms. It formed Hbonds up to ~47 bonds. The neutral surface periclase formed ~9 Hbonds with the nucleotides.

**Figure 8:**
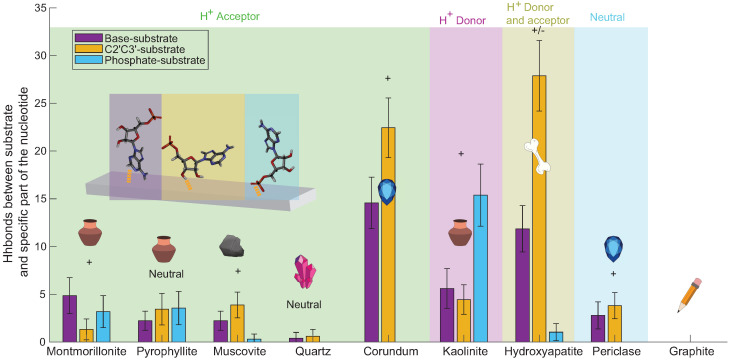
The average number of Hbonds formed between the different substrates and the different parts of the nucleotides at ~5% hydration. Three nucleotide parts were considered: the nucleotides’ base (purple), the hydroxyl $C2^{\prime }$ and/or $C3^{\prime }$ (yellow), and the phosphate (blue). Error bars represent one standard deviation. The color blocks visualize the substrates’ potential to serve as Hbond donors or acceptors. The “+” and “−” represent the positive and negative surface’s charge, respectively. The largest number of bonds was observed for corundum, kaolinite, and hydroxyapatite, between $C2^{\prime }$ and/or $C3^{\prime }$ and the substrate.

We then calculated the number of Hbonds between the phosphate, the base, and the hydroxyl of the nucleotides and the substrates. Figure 8 shows the number of Hbonds between the nucleotide base and the substrate (purple), between the nucleotide sugar and the substrate (to be more specific, between $C2^{\prime }$ and $C3^{\prime }$ of the sugar) in yellow, and the nucleotide phosphate group and the substrate (blue) for all substrates in this study.

Figure 8 shows that not all parts of the nucleotides interact in the same way with the different substrates. Except for montmorillonite, three of the four acceptor surfaces were found to form bonds mainly with the nucleotides’ $C2^{\prime }$ and/or the $C3^{\prime }$. They also created Hbonds with the base of the nucleotides, however, with a lower rate, and they formed a few Hbonds with the phosphate of the nucleotides. Montmorillonite mainly formed Hbonds with the base of the nucleotides. Kaolinite formed 62% of its Hbonds with the phosphate of the nucleotides. Hydroxyapatite mainly formed Hbonds with the $C2^{\prime }$ and/or $C3^{\prime }$ and the base of the nucleotide. It formed only one Hbond with the phosphate of a nucleotide. Periclase formed around the same number of Hbonds with the $C2^{\prime }$/$C3^{\prime }$ and the base of the nucleotides (four and three, respectively), and none with the phosphate.

### 2.5. No Substrate Is a Good Catalyst for the Formation of Pre-Polymers

**Figure 9:**
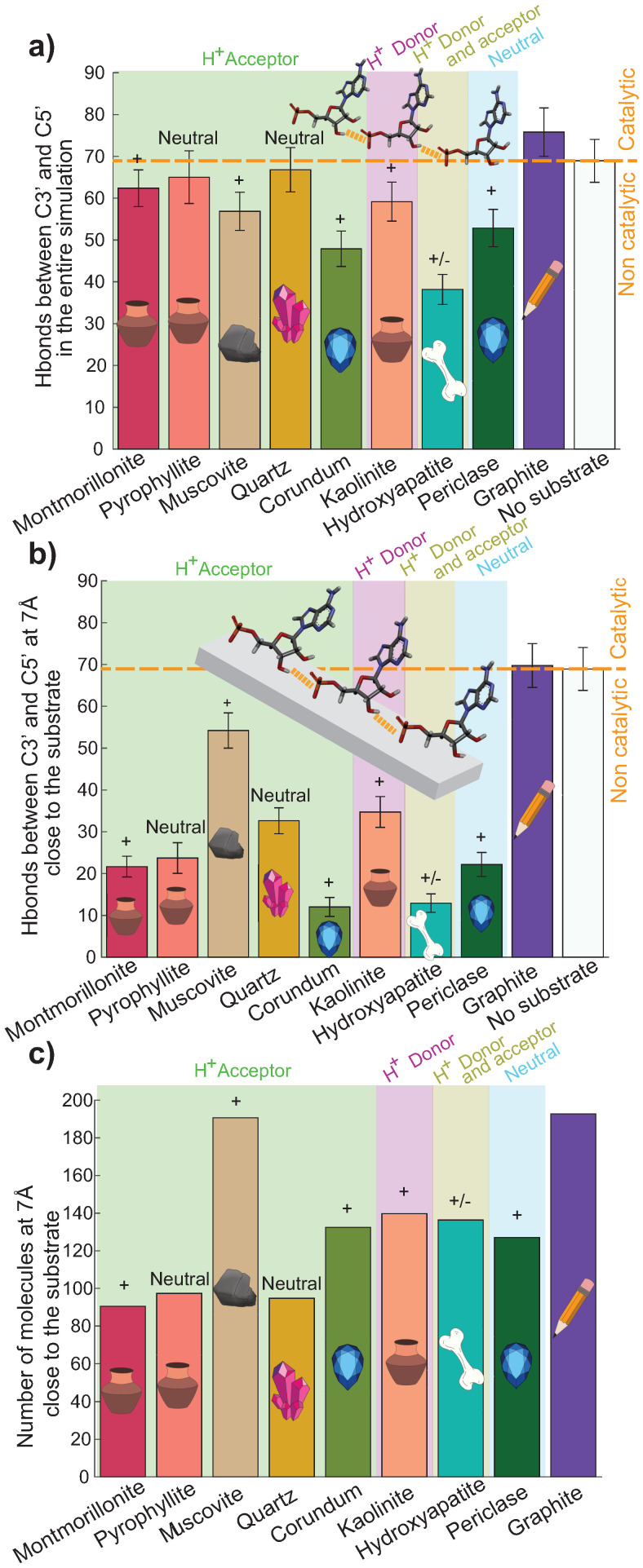
(**a**) The average number of Hbonds formed in the entire box between $C3^{\prime }$ and the phosphate of the nucleotides ($C5^{\prime }$) in the presence of the different substrates. (**b**) The average number of $C3^{\prime }$–$C5^{\prime }$ Hbonds between molecules within a 7 Å distance to each substrate. (**c**) The average number of nucleotides close to the substrate. All results at ~5% hydration, error bars represent one standard deviation. The color blocks visualize the substrates’ potential to serve as Hbond donors or acceptors. The “+” and “−” represent the positive and negative surface’s charge respectively. See text for explanations.

The $C3^{\prime }$ link to the $C5^{\prime }$ is the most critical linkage for the origin of life as it is the bond that links the nucleotides in modern RNA and DNA. We thus calculated the number of Hbonds between those two atoms in the presence of the different substrates. Figure 9a shows the number of Hbonds between $C3^{\prime }$ and $C5^{\prime }$ in the entire simulation box at ~5% hydration. Figure 9b shows the same type of Hbonds, however, only including nucleotides at a distance closer than 7 Å to the surface to check whether the direct proximity of the substrate has an influence on the formation of these pre-polymers. Figure 9c plots the number of nucleotides closer than 7 Å to the surface.

In Figure 9, we also included values for two additional systems. We included graphite in the simulations as an inert substrate, which is not charged nor capable of forming hydrogen bonds with the nucleotides. Graphite thus allowed us to study the effect of a non-interacting surface on pre-polymer formation. We also simulated a nucleotide-only system, i.e., 200 nucleotides, that were dehydrated using the same protocol as in the substrate simulations. By comparing to this simulation, one should be able to determine the efficiency of the different substrates, and if they actually work as catalysts for pre-polymer formation.

As a first finding, the largest number of $C3^{\prime }$–$C5^{\prime }$ Hbonds is found in the presence of the neutral graphite, or in the absence of a substrate, as shown in Figure 9a. Within the given statistics, only montmorillonite, pyrophyllite, and quartz lead to a similar number of Hbonds; the number of Hbonds for the other substrates falls below the standard deviation of the nucleotide-only and graphite systems. All of the other substrates thus seem to inhibit the formation of pre-polymers, which include muscovite, corundum, kaolinite, hydroxyapatite, and periclase.

From the data in Figure 9b, the role of the substrate for pre-polymer formation can be determined, as only nucleotides closer than 7 Å to the surface were selected. The largest number of Hbonds was again observed for the neutral graphite and the nucleotide-only system. Except for muscovite, the number of all bonds was significantly lower than the number of total bonds in part a. This is indicative that the presence of a substrate does not drive bond formation as $C3^{\prime }$–$C5^{\prime }$ Hbonds are primarily formed further away than 7 Å from the substrate. To support this hypothesis, Figure 9c displays the number of nucleotides closer than 7 Å to the substrate. For three substrates (montmorillonite, pyrophyllite, and quartz), more than half the nucleotides are further than 7 Å from the surface. Those substrates do not seem to attract the nucleotides efficiently. The other substrates, corundum, kaolinite, hydroxyapatite, and periclase, seem to be attractive to the nucleotides, however, the substrates do not seem to strongly support the formation of pre-polymers.

### 2.6. Substrate Surfaces Can Align Nucleotide through Interactions

**Figure 10:**
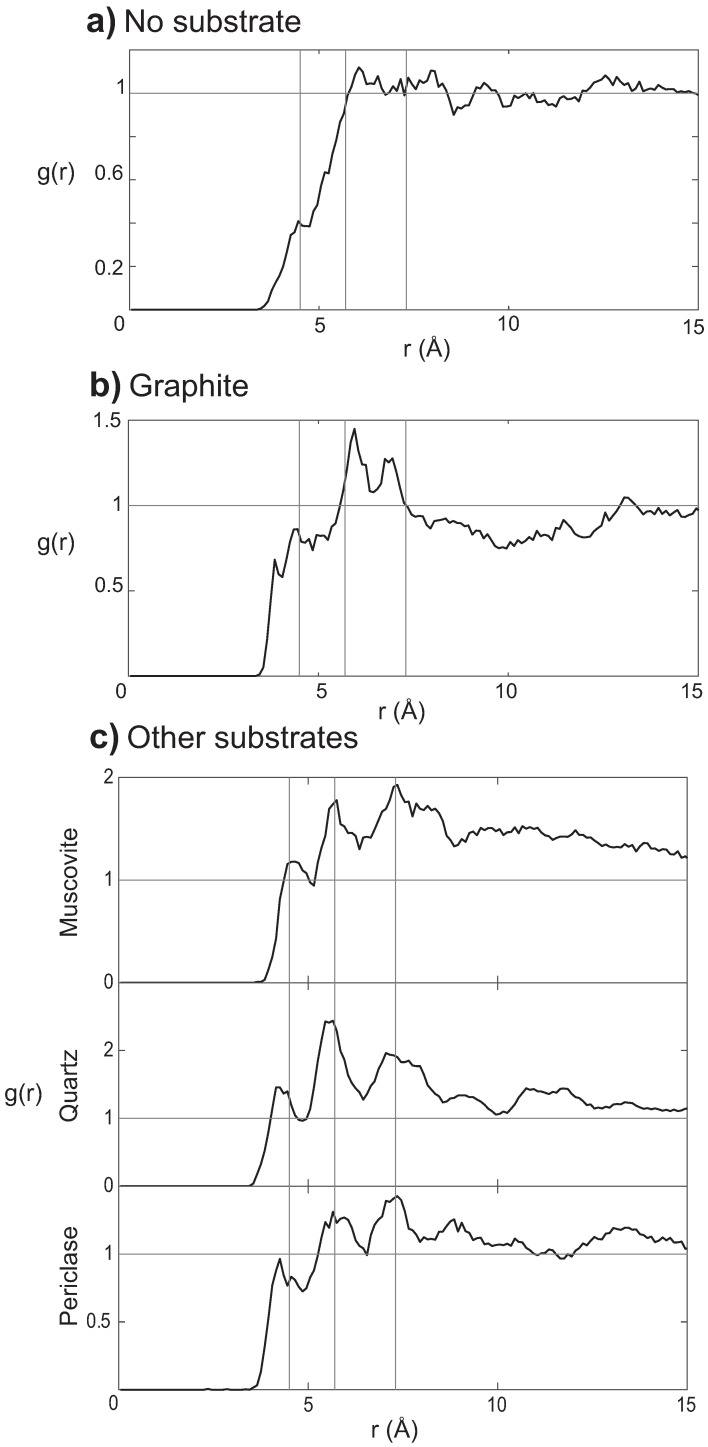
Radial pair distribution function g(r). (**a**) The g(r) of the simulation without substrate at ~5% hydration. (**b**) The g(r) of the nucleotides with graphite at ~5% hydration. Three peaks are distinguished at 4.3 Å, 6 Å and 6.8 Å. (**c**) The g(r) of muscovite, quartz, and periclase as examples. Three peaks can be seen at 4.5 Å, 5.7 Å and 7.3 Å.

It is important to notice that even though substrates do not seem to support the formation of pre-polymers, they serve to organize the nucleotides. The radial pair distribution function g(r) between the $C3^{\prime }$ of the nucleotides has been calculated for all systems, and is presented in Figure 10. No defined peaks are visible in Figure 10a for g(r) of the nucleotide-only system at ~5%. In the presence of graphite, however, in Figure 10b, three peaks can be distinguished at 4.3 Å, 6 Å, and 6.8 Å. g(r) of muscovite, quartz, and periclase are exemplary shown in Figure 10c (the other substrates provided similar results). Distinct peaks were observed here as well, at positions of 4.5 Å, 5.7 Å, and 7.3 Å, slightly shifted in comparison to graphite.

**Figure 11:**
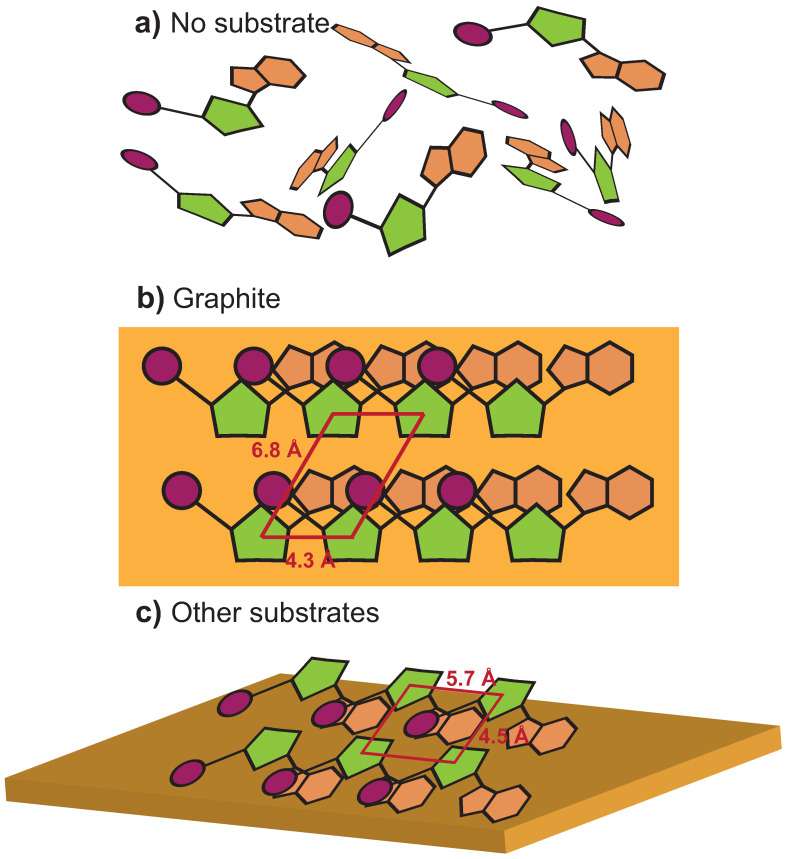
Organization of the nucleotides in the simulations. (**a**) The non-organization of the simulation without substrate at ~5% hydration. (**b**) The nucleotide organization with graphite at ~5% hydration. The nucleotides are laying down on the substrates. The unit cell is a parallelogram with *a* = 6.8 Å and *b* = 4.3 Å. (**c**) The nucleotide organization in presence of the other substrates studied at ~5% hydration. The unit cell is rectangular with *a* = 5.7 Å and *b* = 4.5 Å.

**Figure 12:**
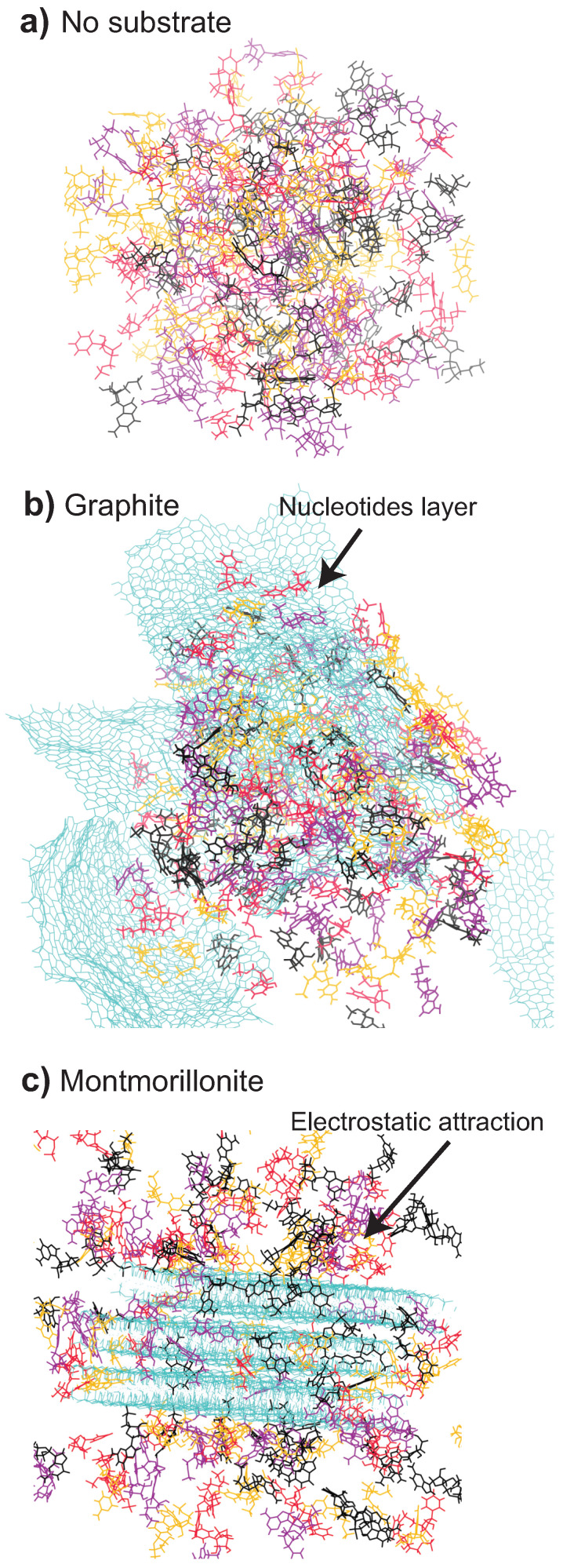
Snapshot of the different simulations at ~5% hydration. In purple is AMP, in red—UMP, in black—GMP, and in orange—CMP. The substrates are shown in cyan. (**a**) Snapshot of the simulation without substrate. (**b**) The graphite simulation. Nucleotides formed a flat layer on the sheets of graphite. (**c**) Montmorillonite simulation as an example. Nucleotide phosphates are attracted to the substrate due to the different charges.

These graphs reveal an organization of the nucleotides in the presence of substrates, as sketched in Figure 11. Without a substrate in Figure 11a, the nucleotides are completely disordered in the water phase. This can also be seen in the MD simulations, in the snapshot in Figure 12a. In the presence of graphite (at ~5% hydration) in Figure 11b, the nucleotides align flat on the substrates, forming a hexagonal unit cell with side lengths *a* = 6.8 Å, and *b* = 4.3 Å, and angle α = 120${}^{\circ }$. Evidence for this type of organization is found in the simulations, as shown in Figure 12b, where nucleotides have a tendency to form flat layers on the graphite sheets.

In the presence of charges, there is an attraction between certain molecular groups in the nucleotides and the substrate, and in order to minimize this electrostatic energy, the nucleotides are no longer aligning flat on those substrates. The electrostatic interactions between the phosphate and the base, for instance, would lead to an arrangement as in Figure 11c, where the nucleotides are slightly tilted, and the different layers can move more closely and become more densely packed. This type of organization can be observed in the MD snapshots in Figure 12c. When the nucleotides are slightly tilted, a second layer of nucleotides can slide under the first layer. The corresponding unit cell is rectangular with *a* = 5.7 Å, and *b* = 4.5 Å.

## 3. Discussion

Our goal was to find the physical properties of the substrates that support the formation of RNA polymers in a prebiotic environment in the context of the wet–dry cycling theory. To do so, we used Molecular Dynamics simulations using nine different substrates with different charges and hydrogen bonding potential. Some researcher used MD simulations to study wettability of clay [119]. They found that kaolinite is overall hydrophilic and that the OH-groups on the *O* surface can form unstable hydrogen bonds with water. MD simulations were also studied for the polymerization of peptides. Quartz and clays were used in this research [120,121,122,123,124,125,126,127,128,129,130]. Other groups studied the formation of nucleotides using MD simulations [131,132,133], but to our knowledge, these are the first simulations to study the interaction between nucleotides and substrates. Our initial hypothesis was that the presence of substrates has a catalytic effect on the formation of pre-polymers that show $C3^{\prime }$–$C5^{\prime }$ hydrogen bonds between nucleotides.

Ferris, Ferris et al. [66,67,68,134], and Huang and Ferris [77] used montmorillonite clay as a catalyst to promote RNA polymerization. Using activated nucleotides, they were able to form RNA polymers up to 40 mers. More recently, rock glasses have been used to convert ribonucleosides 5′-triphosphates into RNA long of 9–150 mers on average [135].

Substrates are also used for template-directed synthesis. Holm et al. [73] used iron oxide hydroxide polymorph to concentrate and form a longer chain of polymers. They again explain the adsorption of the nucleotides onto the surface by electrostatic interaction between the negative charge of the phosphate and the positive surface, as underlined in the introduction. Using hydroxyapatite, Acevedo et al. [74] were able to form polymers of up to 20–25 nucleotides after only one cycle of reactivation and ligation using a template. The length of the polymers increased when increasing the number of reactivation–ligation cycling.

The implicit effect of the concentration is the stabilization of biomolecules by the surfaces. Biondi et al. [136,137] emphasized that opal, alkaline carbonate, and sulfate minerals also stabilized polymers, in addition to concentrating RNA. Along with stabilization, it has been shown that minerals protect biomolecules from environmental damage. Huang and Ferris [77] have established that montmorillonite protected nucleotides from hydrolysis. Other research has shown that polymers could have been protected from UV radiation by diver minerals on primitive Earth [75,96,138].

We found that the charge of the substrates impacts the attraction and the binding of the nucleotides onto the substrate. Positively charged substrates showed a tendency to attract the negatively charged phosphate group. The substrates that work as hydrogen bond donors can form hydrogen bonds with the phosphates. This number increases when water is removed from the system. Overall, the nucleotides behaved as expected while in contact with the different substrates.

The next step was to calculate the number of pre-polymers in different environments. The surprising result is that none of the charged and hydrogen bond potential substrates has been found to be a catalyst when compared to the nucleotide-only system. The three substrates that give the best results had very different properties. Muscovite is a positively charged hydrogen acceptor, kaolinite is a positively charged hydrogen donor, and quartz is neutral and a hydrogen acceptor. Hydroxyapatite, which is a donor and a hydrogen acceptor substrate, with positive and negative charges, formed an important number of Hbonds with the nucleotides, but the nucleotides did not form a significant number of $C3^{\prime }$–$C5^{\prime }$ Hbonds in the presence of this substrate.

Our simulations of graphite provide the deepest insight into the role of mineral surfaces for pre-polymerization. Unlike the other minerals we simulated, graphite does not have any charges, or the ability to form Hbonds with the nucleotides, but it was found to strongly promote the formation of $C3^{\prime }$–$C5^{\prime }$ Hbonds. In fact, graphite was as efficient as the nucleotide-only system without a substrate to promote pre-polymers’ formation. Graphite was already studied in the origin of life context by Sowerby et al. [110,139]. They showed that xanthine formed monolayers on graphite at a solid–liquid interface. They also observed a periodic lattice structure. Other papers highlight the position of different bases on graphite. By measuring the adsorption isotherm, they also found that some bases are adsorbed on the surface better than others. Therefore, we conclude that inert substrates, such as graphite, or the complete absence of a substrate are likely preferred for forming pre-polymers on primitive Earth. While it has been speculated that a substrate can help to organize and confine nucleotides, the presence of a substrate at the same time was found to reduce the number of interactions between nucleotides.

While it is straightforward to set up a system that contains nucleotides and water, and successively reduces the number of water molecules to mimic dehydration, the question remains how relevant such a system is. Such a system mimics supersaturated water, maybe droplets, but it is a system that is almost dried out. We suggest that those systems could have been present on primitive Earth via the presence of geysers for example. Indeed, springs or geysers can produce very fine water droplets that dry quickly when exposed to air, heat, and the Sun, depending on the size of the droplets. During this drying process, pre-polymers can form in the first step, and phosphodiester bonds between the nucleotides to form RNA polymers can be formed in a second step when additional energy in the form of heat is provided. The dried pellet can potentially become part of a pond where the RNA can later be encapsulated. In case amphiphilic molecules are present in the droplet simultaneously as nucleotides, the RNA polymers could be encapsulated in liposomes in the same process [16].

What is the possibility of having such droplets that linger in the air for long enough (several seconds) for those reactions to occur? Several forces affect the length of time of a droplet in the air: gravity ($F_{g}=\rho_{water}V_{droplet}g$), the force of buoyancy ($F_{B}=\rho_{air}V_{droplet}g$), and the viscous resistance of air ($F_{Stokes}=6\pi \nu rv$), see, for instance, [140]. The terminal sedimentation velocity of droplets is then calculated to $v_{t}=2r^{2}g\left(\rho_{water}-\rho_{air}\right)/\left(9\eta \right)$, where *r* is the droplet’s radius, *g* is the gravity constant, η is the viscosity of the air, $\rho_{water}$ is the density of water and $\rho_{air}$ is the density of air. Assuming that such a droplet would fall from a certain height, the time of the fall is calculated as $t_{fall}=H/v_{t}$. At the same time, evaporation limits the lifetime of a water drop by $t_{life}=2r^{2}/\left(q_{0}\Delta T\right)$, where *r* corresponds to the radius of the droplet, ΔT is the difference between ‘dry-bulb’ and ‘wet-bulb’ temperature (the ‘wet-bulb’ temperature corresponds to the temperature where the drop is cooling down due to evaporation), and $q_{0}$ (in $\mu m^{2}s^{-1}K^{-1}$) depends on the ambient conditions and the liquid properties [140]. At 20 °C and 50% RH, Δ*T* is equal to 6.1 °C and $q_{0}$ is equal to 89.84 μm${}^{2}$s${}^{-1}$K${}^{-1}$ [140].

**Figure 13:**
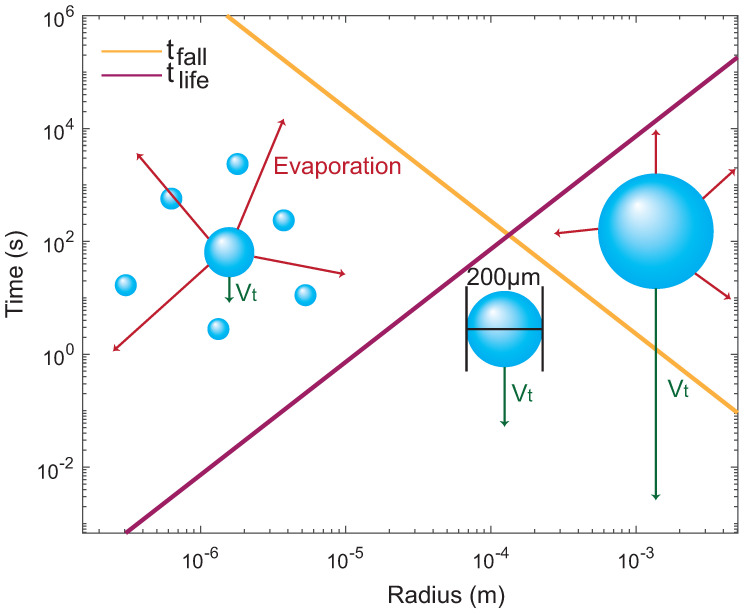
Graph of the time of a droplet in the air (to sediment from a height of 5 m, yellow), and the lifetime of a droplet limited by evaporation (purple) as a function of the droplet radius. Droplets around *r* = 100 μm can live for ~100 s, which would provide enough time for pre-polymers and polymers to form.

In Figure 13, $t_{fall}$ is plotted (for a height of 5 m, yellow) together with the lifetime of a droplet (at 20 ${}^{{}^{\circ}}$C and 50% relative humidity, purple). Smaller droplets would stay in the air long enough for pre-polymerization and polymerization to happen; however, they will evaporate too quickly. Larger droplets on the other hand would live longer but they would not stay in the air long enough but quickly fall to the ground. The intersection of the two lines provides an estimate of an optimal droplet size of about ~200 μm in diameter, which corresponds to a volume of 0.0042 μL. Such droplets would live for ~100 s, which would likely leave enough time for pre-polymerization and polymerization to occur [15].

It is important to note that the literature emphasizes the importance of substrates and their catalytic properties when reactions are carried out in aqueous environments. However, as the synthesis of RNA from monomers is thermodynamically impossible in the presence of water [141], these studies typically involve activated nucleotides, and RNA polymerization is observed in the presence of substrates. We found that non-activated nucleotides first start to interact in aqueous environments via hydrogen bonds, however, hydrogen bond formation becomes significant only when water is removed, and the system is dehydrated. In the absence of water, we then found that substrates may have played a secondary role in the formation of pre-polymers and potentially also in the origin of life. Substrates do play a role in the organization of nucleotides; however, because of their interaction with the surface, nucleotides are limited in their availability to interact with each other. Our results suggest that non-interactive surfaces, or even the absence of substrates, are likely best for bond formation while drying the system in an origin of life context.

## 4. Conclusions

We used all-atom Molecular Dynamics computer simulations to investigate the effect of substrates on the formation of $C3^{\prime }$–$C5^{\prime }$ hydrogen bonds between the four nucleotides in RNA (AMP, UMP, CMP, GMP). These hydrogen-bonded pre-polymers have been suggested to be the precursors of phosphodiester bonded RNA-polymers. The nine substrates in this study included three clay minerals, one mica, one phosphate mineral, one silica, and two metal oxides. We also included graphite, a non-charged and non-hydrogen bonding substrate. Wet–dry cycles were simulated by slowly removing water molecules from the simulations. No substrate was found to be a good catalyst for the formation of hydrogen-bonded pre-polymers. The highest number of $C3^{\prime }$–$C5^{\prime }$ hydrogen bonds between nucleotides was found in the presence of graphite or in the absence of a substrate, in a nucleotide-only system. While confinement and dehydration seem to be mainly responsible for hydrogen bond formation, substrate interactions were found to reduce the interactions between nucleotides in all cases, independent of their charge and ability to form hydrogen bonds with the nucleotides. The findings suggest that small supersaturated droplets may be an efficient way to produce pre-polymers. Geysers and springs could have produced these droplets in an early-Earth environment.

## 5. Materials and Methods

### 5.1. Substrates

Nine different substrates were prepared using the CHARMM-GUI nanomaterial modeler [115,116,117] and are listed in Table 1. All substrates had a size of 40 Å × 50 Å × 30 Å.

Some of the substrates can be found in granitic igneous rocks (specific magmatic rocks), in meteorites, and in Ur-minerals (mineral form from presolar grain) [105]. Clay surfaces have a grain size of <2 μm. When wet, clays tend to be more ductile and they harden when dry. Montmorillonite is composed of layers that include one sheet of alumina between two sheets of silica linked through oxygen atoms. Each layer has a thickness of ~1 nm, while the interlayer has a thickness from 0 to 4 nm. Sodium or calcium ions can be present in between the layers, as well as water. Montmorillonite can swell when water is incorporated between the layers. The overall charge of the montmorillonite surface is negative but it is compensated with the absorption of cation around the edges to maintain balance [95]. Montmorillonite used in the simulations has a three repeating unit, each having a thickness of ~7 Å, with an interlayer spacing of ~4 Å. Kaolinite is another type of clay. One kaolinite layer comprises one sheet of silica and one sheet of alumina, linked together via oxygen atoms [95]. Its interlayer size is typically 0.7 nm. No ions are found between the layers, so kaolinite has a low exchange capacity. Layers are linked together via hydrogen bonding. Kaolinite used in the simulation is composed of five repeating units. Each layer has a thickness of ~4.5 Å and an interlayer spacing of ~2 Å. Pyrophyllite’s layers have the same thickness and the same organization as montmorillonite (Silica-Alumina-Silica); however, cations are not present between the layers. Layers are neutral and held together with weak van der Waals bonds [95,142]. This clay is flexible but not elastic. Pyrophyllite presented in this study is composed of four repeated units, with a thickness of ~6 Å and an interlayer distance of ~2.5 Å.

Muscovite is also composed of repeating units. Each unit is composed of three sheets, two silica sheets, and one alumina sheet in between. Potassium is the cation present in between the layers, but mica minerals have little or no exchangeable water. Muscovite’s layers are flexible and elastic. The silica layer has a net negative charge [95]. In our study, muscovite is composed of three layers, with a thickness of ~6.5 Å and an interlayer of ~5 Å with potassium ions in between the layers.

Hydroxyapatite is a phosphate mineral composed of PO${}_{4}{}^{3}{}^{-}$ groups bonded laterally through Ca${}^{2}{}^{+}$[95]. Hydroxyapatite does not have a uniform hydrogen property throughout its surface because of the atoms’ organization in the minerals. In the quartz mineral (SiO${}_{2}$), each silica is held together via four oxygen atoms. Quartz structure is open so it can be incorporated large cations such as Ca${}^{2}{}^{+}$, Na${}^{2}{}^{+}$, K${}^{+}$[95]. Quartz was also used as a substrate in the origin of life research [27,28,29,30]. Quartz is formed of just one block of material. An oxide is a group of minerals that include natural compounds in which oxygen anions (O${}^{2}{}^{-}$) are combined with one or more metals. Strong ionic bonds maintain the atoms together in oxide minerals [95]. All of these minerals do not contain anionic groups. In the corundum case (Al${}_{2}$O${}_{3}$), Al${}^{3}{}^{+}$ is surrounded by six oxygens, and four bonds can radiate from an oxygen [95]. For periclase (MgO), each oxygen is shared between six Mg-O (and not just four Al-O); therefore, MgO does not show cation vacancies [95]. Corundum and periclase are also just composed of just one block of material.

Graphitic carbon is a general term given to solid carbonaceous compounds whose structure is based on 6-fold rings of carbon atoms [101]. Graphite sheets are bonded to each other with van der Waals interaction [95]. Six sheets of graphite were used in our simulations.

### 5.2. Molecular Dynamics Simulations

Substrates were then run in presence of 50 molecules of adenosine monophosphate (AMP), uridine monophosphate (UMP), cytidine monophosphate (CMP), and guanosine monophosphate (GMP) each. Molecules were taken from the CHARMM-GUI archive-small molecules library using CHARMM-GUI multi component assembler [115,116,117]. The size of the simulation box was 85 Å${}^{3}$. Simulations were run with GROMACS 2016.3 on MacSim, a GPU-accelerated computer workstation [114]. This computer was equipped with a 40 Core central processing unit (CPU, Intel(R) Xeon(R) CPU E5-2630 v4 @ 2.20GHz), 130 GB random-access memory (RAM), and three graphic processing units (GPU, 2 NVIDIA 1080 TDI + 1 GeForce GT 730).

The systems were first energy minimized and then equilibrated for 100 ps using an NVT ensemble before being simulated for 10 ns at 100% hydration. All simulations used a 2 fs time step, a periodic boundary cutoff of 1.2 nm, the particle-mesh Ewals method using a real-space cutoff of 1.2 nm, fourth-order interpolation, and 0.16 nm grid spacing to solve for long-range electrostatics. The parallel LINCS (P-LINCS) algorithm was used to determine bond constraints [143]. Temperature and pressure were maintained at 303 K and 1.0 bar using a Nosé–Hoover thermostat [144,145,146] at 30 ${}^{{}^{\circ}}$C (τ = 1.0 ps) and Parrinello–Rahman isotropic weak coupling (τ = 1 ps) [147]. All analyses were performed on the entire simulations.

Water was removed successively during the simulations to mimic dehydration and a wet–dry cycle. For each step of the dehydration protocol, the next simulation, a new index file was created by selecting all the molecules except the water molecules. The last frame from the previous simulation was extracted using the GROMACS command *gmx trjconv*. The last frame was solvated and a new system was created using the GROMACS command *gmx solvate*. The new system was then energy minimized, equilibrated, and run in the same condition than 100% hydration. The systems were run for 15 ns. The number of water molecules per simulation is listed in Table S1 (in the Supplementary Material) for the system with substrates and in Table S2 (Supplementary Material) for the nucleotides-only system. Hydrogen bond analysis was performed using VMD built-in algorithms.

## Figures and Tables

**Table 1:** List of all substrates in this study and their composition.

Material	Mineral	Formula
Clay	Montmorillonite	(K, Na)${}_{n}$[Si${}_{4}$O${}_{8}$][Al${}_{2n}$Mg${}_{n}$O${}_{2}$(OH)${}_{2}$]
	Kaolinite	Al${}_{2}$Si${}_{2}$O${}_{5}$(OH)${}_{4}$
	Pyrophyllite	Al${}_{2}$Si${}_{4}$O${}_{10}$(OH)${}_{2}$)
Mica	Muscovite	(KAl${}_{2}$(AlSi${}_{3}$)O${}_{1}0$(OH)${}_{2}$)
Phosphate minerals	Hydroxyapatite	Ca${}_{5}$(PO${}_{4}$)${}_{3}$(OH)
Silica	α-quartz	SiO${}_{2}$
Metal oxides	Corundum	Al${}_{2}$O${}_{3}$
	Periclase	MgO
Carbonaceous material	Graphite	C

## Data Availability

All data including MD files and trajectories are available upon request.

## Supplementary Materials

The following supporting information can be downloaded at: https://www.mdpi.com/article/10.3390/life13010112/s1, Table S1: Percentage of hydration and number of water molecules for all simulations containing a substrate; Table S2: Percentage of hydration and water molecules for the nucleotides-only system.
