# Supplementary material for: The Formation of RNA Pre-Polymers in the Presence of Different Prebiotic Mineral Surfaces Studied by Molecular Dynamics Simulations
Source: Life (Basel). 2022 Dec 30;13(1):112. doi: 10.3390/life13010112 (PMC9860743; doi:10.3390/life13010112)
Supplement: Supplementary file 1 [file life-13-00112-s001.zip › life-2087913-supplementary.pdf]

SUPPLEMENTARY MATERIAL TO: THE FORMATION OF RNA PRE-POLYMERS IN THE PRESENCE OF DIFFERENT PREBIOTIC MINERAL SURFACES STUDIED BY MOLECULAR DYNAMICS SIMULATIONS

Percentage of hydration (%)	Total number of water molecules	Approximate number of water molecules per nucleotide
100	12263-12878	61-64
85.4-89.7	10170-11000	51-55
75.3-78.9	8949-9680	45-48
61.3-69.7	7175-8470	36-42
53.5-59.2	6258-7260	31-36
40.8-51.6	5217-6050	26-30
31.4-41.3	4019-4840	20-24
24.2-31.1	3094-3630	15-18
18.8-20.7	2399-2420	12
9.0-10.3	1210	6
4.6-5.2	605	3

Supplementary Material, Table S1. Percentage of hydration and number of water molecules for all simulations containing a substrate.

Percentage of hydration (%)	Total number of water molecules	Approximate number of water molecules per nucleotide
100	15436	77
71.3	11014	55
61.9	9549	48
54.8	8462	42
47.6	7344	37
34.6	5340	27
28.8	4452	22
23.2	3579	18
16.2	2500	12
6.5	1000	5
3.2	500	3

Supplementary Material, Table S2. Percentage of hydration and water molecules for the nucleotides-only system.
